# Shift work, low-grade inflammation, and chronic pain: a 7-year prospective study

**DOI:** 10.1007/s00420-020-01626-2

**Published:** 2021-02-07

**Authors:** Jan Olav Christensen, Kristian Bernhard Nilsen, Laila Arnesdatter Hopstock, Ólöf Anna Steingrímsdóttir, Christopher Sivert Nielsen, John-Anker Zwart, Dagfinn Matre

**Affiliations:** 1 National Institute of Occupational Health, Oslo, Norway; 2 Oslo University Hospital, Oslo, Norway; 3 UiT, The Arctic University of Norway, Tromsø, Norway; 4 Norwegian Institute of Public Health, Oslo, Norway

**Keywords:** Working time, Chronic widespread pain, Number of pain sites, C‐reactive protein, hs‐CRP, Shift work

## Abstract

**Objectives:**

We investigated prospective associations of shift work with chronic pain and C‐reactive protein (CRP), an indicator of inflammation. Furthermore, we elucidated CRP as a possible mediator and/or moderator of effects of shift work on pain.

**Methods:**

Data from a 7 years follow‐up study were analyzed (*N* = 2323). Shift work and chronic pain of “neck/shoulder”, “arm/hand”, “upper back”, “low back”, “hip/leg/feet”, and “other regions” were measured by questionnaires. “Chronic widespread pain”, “number of chronic pain sites”, and “any chronic pain” were computed. CRP was measured in serum samples. Logistic and Poisson regressions were conducted. Mediation was assessed by casual mediation analyses and moderation by the Relative Excess Risk due to Interaction (RERI).

**Results:**

Shift work was not associated with any chronic pain variable and no mediation was detected. CRP was associated with low back pain, hip/leg pain, and “number of pain sites”, and also with the combination of shift work and CRP of 1–2.99 mg/L (compared to: no shiftwork and CRP < 1). Additionally, shiftwork and CRP 1–2.99 mg/L was associated with risk of “any chronic pain” (OR: 1.76, 95% CI: 1.12, 2.85), which was not associated with CRP alone. Moderation analyses suggested the risks for “any chronic pain” and “number of pain regions” increased when individuals with elevated CRP worked shifts—beyond what the separate effects of CRP and shift would suggest.

**Conclusions:**

We found no evidence of shift work in general affecting CRP or chronic pain. However, shift work and elevated CRP combined may influence chronic pain.

**Supplementary Information:**

The online version contains supplementary material available at 10.1007/s00420-020-01626-2.

## Introduction

Shift work and non‐standard working hours have been linked to health problems and health behaviours such as sleep loss, accidents, weight gain, type 2 diabetes, coronary heart disease, stroke, and cancer (Kecklund and Axelsson 2016). Musculoskeletal pain has also been found to be more common among shift workers, although the mechanisms that may explain the association remain poorly understood (Caruso and Waters 2008). One possibility is that shift work promotes inflammation (Puttonen et al. 2011; Sookoian et al. 2007), which may increase the risk and severity of pain (Briggs et al. 2013). Low-grade inflammation may slightly increase levels of unspecific markers of inflammation like C‐reactive protein (CRP). Hence, the current study aimed to examine the long‐term (7 years) association of shift work with several musculoskeletal pain complaints as well as to determine whether levels of CRP in shift workers could account for or exacerbate any such association.

As organizations in contemporary society seek to improve efficiency and productivity in the emerging “24‐hour society”, many individuals are required to work outside of a traditional “9‐to‐5” business day (Rajaratnam and Arendt 2001; Grandner 2017). In Norway, approximately 25% of working age adults work shifts (Tynes et al. 2018). The requirement to work non‐standard hours can be at odds with biological and social adaptations to the 24 h cycle of light and darkness, and may thus influence sleep and restitution as well as physical, mental, and social functioning. The term circadian stress has been coined to collectively denote physiological, behavioral, and psychosocial consequences of disturbances to the human circadian rhythm (Puttonen et al. 2010). With regards to pain, studies have suggested night work to be associated with increased sensitivity to pain (Matre et al. 2017) and increased risk of some types of pain complaints (Katsifaraki et al. 2019). In Norway, 25% of the working population experience work-related neck and shoulder pain, and 15% report low back pain (Tynes et al. 2018). Hence, if shift work contributes to musculoskeletal health, it may represent a substantial impact on public health that may continue to grow with impending societal changes.

Mechanisms that may explain associations of shift work with pain remain elusive. However, for the link between shift work and cardiovascular disease (CVD), some suggestions have been made that may be relevant for pain complaints as well. Several systematic reviews have connected shift work with CVD, often based on the notion of pro‐inflammatory sleep disruptions as a consequence of working hours, particularly working night shifts (Kecklund and Axelsson 2016; Vyas et al. 2012; Torquati et al. 2018). Previous studies have reported that sleep disturbances influenced levels of CRP specifically (Meier-Ewert et al. 2004). Other risk factors of CVD that may influence inflammation in shift workers include smoking, physical inactivity, unhealthy dietary choices, irregular eating, alcohol use, and more generally, low socioeconomic status (Bøggild and Knutsson 1999; Kecklund and Axelsson 2016). Some studies have also reported elevated levels of inflammation in shift workers compared with day workers (Puttonen et al. 2011; Sookoian et al. 2007; Skogstad et al. 2019), and extended work shifts that involve night shifts have been observed to correlate with elevated CRP (Faraut et al. 2013). In addition to the possible impact of sleep and health behaviors, psychoneuroendocrine responses to disrupted private and social life when working non‐standard working hours may also promote inflammation (Johnson et al. 2013; Bøggild and Knutsson 1999).

In addition to affecting health directly, another explanation for the elevated risk of morbidity in shift workers may be that shift work potentiates the vulnerability to traditional risk factors. A recent study found shift work to be associated with an exacerbation of the adverse impact of established risk factors on myocardial infarction (Hermansson et al. 2019). Moreover, an animal study showed that sleep deprivation prior to an inflammatory challenge was associated with significantly enhanced mechanical sensitivity tested by the von Frey test (Vanini 2016), suggesting the combination of poor sleep and inflammation causes more pain than one would expect based on the effects of each condition separately. As sleep, and particularly slow‐wave sleep, can attenuate pro‐inflammatory immune responses, increased sleep is often an adaptive first response to illness (Ranjbaran et al. 2007). Hence, shift work could play a part in maintaining or exacerbating chronic pain by not allowing necessary adaptations, such as sufficient sleep and restorative rest, to take place.

The current study sought to elucidate the abovementioned concerns by utilizing a large population sample to determine whether working shifts was associated with musculoskeletal pain complaints 7 years later. Furthermore, we examined the extent to which such associations were mediated or moderated by the level of CRP.

## Materials and methods

### Participants and design

We used data from the Tromsø Study, a longitudinal population‐based cohort study carried out in the municipality of Tromsø, Norway (Jacobsen et al. 2012). Seven surveys have been conducted between 1974 and 2016, to which total birth cohorts and random samples have been invited. Data collection includes questionnaires and interviews, biological sampling and clinical examinations. The current analysis is prospective, utilizing data from two waves of the study, Tromsø 6 (2007–2008, *N* = 12,984, participation 66%) (baseline) and Tromsø 7 (2015–2016, *N* = 21,083, participation 65%) (follow‐up). We included women and men below 70 years of age at baseline that participated at both time points, were in full-time employment at both time points, and had data on all variables of the analyses. The effective sample size was 2323 (Table 1).

**Table 1:** Baseline descriptives of the study sample (*N* = 2323)

	*N*	%	Mean	SD
Female	1279	55.1	*–*	*–*
Age	*–*	*–*	46.0	6.3
Education	*–*	*–*	*–*	*–*
Primary/secondary school	228	9.8	*–*	*–*
Technical/vocational school, 1–2 years senior high school	478	20.6	*–*	*–*
High school diploma	226	9.7	*–*	*–*
College/university < 4 years	568	24.5	*–*	*–*
College/university > 3 years	823	35.4	*–*	*–*
Worked shifts previous three months	295	12.7	*–*	*–*
*CRP*	*–*	*–*	1.5	2.9
< 1	1204	51.8	*–*	*–*
1–2.99	831	35.8	*–*	*–*
2.99–10	250	10.8	*–*	*–*
> 10	38	1.6	*–*	*–*
Chronic pain				
Neck pain				
None	1533	66.0	*–*	*–*
Mild or severe	790	34.0	*–*	*–*
Arm pain				
None	1870	80.5	*–*	*–*
Mild or severe	453	19.5	*–*	*–*
Upper back pain			*–*	*–*
None	1984	85.4	*–*	*–*
Mild or severe	339	14.6	*–*	*–*
Low back pain				
None	1757	75.6	*–*	*–*
Mild or severe	566	24.4	*–*	*–*
Hip and leg pain				
None	1819	78.3	*–*	*–*
Mild or severe	504	21.7	*–*	*–*
Other pain				
None	2231	96.0	*–*	*–*
Mild or severe	92	4.0	*–*	*–*
Number of chronic pain sites				
0	1117	48.1	*–*	*–*
1	440	18.9	*–*	*–*
2	329	14.2	*–*	*–*
3	216	9.3	*–*	*–*
4	138	5.9	*–*	*–*
5	52	2.2	*–*	*–*
6	31	1.3	*–*	*–*
Any chronic pain				
No	1117	48.1	*–*	*–*
Yes	1206	51.9	*–*	*–*
Chronic widespread pain				
No	2234	96.2	*–*	*–*
Yes	89	3.8	*–*	*–*

The current study was approved by the Regional Committee for Ethics in Medical Research (REC nr. 2016/1997) and the Norwegian Data Protection Authority. The participants supplied written informed consent, and the study was conducted in accordance with the Declaration of Helsinki.

### Measures

#### Outcomes

Chronic musculoskeletal pain was assessed by questionnaire with six items referring to the following body regions: “neck/shoulder”, “arm/hand”, “upper back”, “low back”, “hip/leg/feet” and “other regions”. Participants were asked whether they had suffered from pain and/or stiffness in muscles and joints in these body regions that lasted for three or more consecutive months during the previous year. Response options were: “no complaints”, “mild complaints” and “severe complaints”. For the current analyses, when single pain sites or number of chronic pain sites were the outcomes, “mild complaints” and “severe complaints” were collapsed to reflect “mild or severe chronic pain”. Chronic widespread pain was defined in accordance with Skarpsno et al. (2019) as mild or severe complaints in all three anatomic regions of (1) neck/shoulder/upper back/lower back, (2) arm/hand, and (3) hip/leg/feet, as well as severe pain in at least one these regions. This definition approximates the 1990 criteria of the American College of Rheumatology of pain being present in the axial skeleton, left and right sides of the body, and above and below the waist (Wolfe et al. 1990), although participants were not asked to report whether pain was present in both sides of the body. “Number of chronic pain sites” was also used as outcome, by counting the number of sites for which a respondent reported mild or severe chronic pain. Finally, “any chronic pain” was defined as a subject reporting mild or severe chronic pain from at least one anatomic site.

### Exposure

Shiftwork was measured by questionnaire, with the single item “Have you had shift work during the previous 3 months?”, with optional answers “Yes” and “No”.

### Mediator/moderator

CRP was measured by high sensitivity (hs) tests in non-fasting serum samples. All analyses were conducted by an ISO-certified laboratory (Department of Laboratory Medicine at the University Hospital of North Norway, Tromsø, Norway; NS-EN ISO 15189: 2012), in accordance with the manufacturer’s protocol (http://labogids.sintmaria.be/sites/default/files/files/crphs_2019-01_v11.pdf). Blood samples were centrifuged for 10 min at 20 °C at speeds of 2000 g (baseline) and 2500 g (follow-up). Samples were stored refrigerated at 4 °C, and kept in room temperature for 30 min prior to centrifuging.

CRP was categorized as: “ < 1.00 mg/L”, “1.00–2.99 mg/L” and “ ≥ 3.00 mg/L”. These levels have been associated with different levels of disease risk (Pearson et al.,^2003^; Kushner et al. 2006). Categorization of CRP was done for three reasons: (1) to assess effects at each of the three clinically relevant levels of CRP, (2) to highlight any potential non-linear associations (e.g., thresholds where only high levels exhibit effects), and (3) to investigate effects of combinations of different levels of CRP with shift work or no shift work. However, as this may constitute information loss, analyses of pain regressed on CRP were also run with continuous CRP to determine if results were substantively different. Small differences were revealed, with continuous CRP exhibiting fewer statistically significant effects than the categorized measure. This suggests that important information may have been added rather than lost by categorizing the variable.

Participants with hsCRP values above 10 mg/L were excluded, as analyses might be affected by including participants possibly suffering acute inflammatory conditions. This group was small, with only 38 participants at baseline (see Table 1), and no subjects exhibiting such high levels at both time points. However, as a sensitivity check analyses involving CRP were rerun with these subjects included, and revealed no substantive differences in results.

### Control variables

All analyses included sex, age, and educational level as control variables. Education was assessed by the item “What is your highest level of education?”, with optional answers “primary or secondary school”, “technical school, vocational school or 1–2 years senior high school”, “high school”, “university/university college < 4 years”, and “university/university college > 3 years”.

### Statistical analyses

**Fig. 1:**
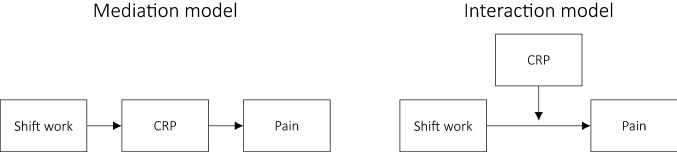
Conceptual models

**Fig. 2:**
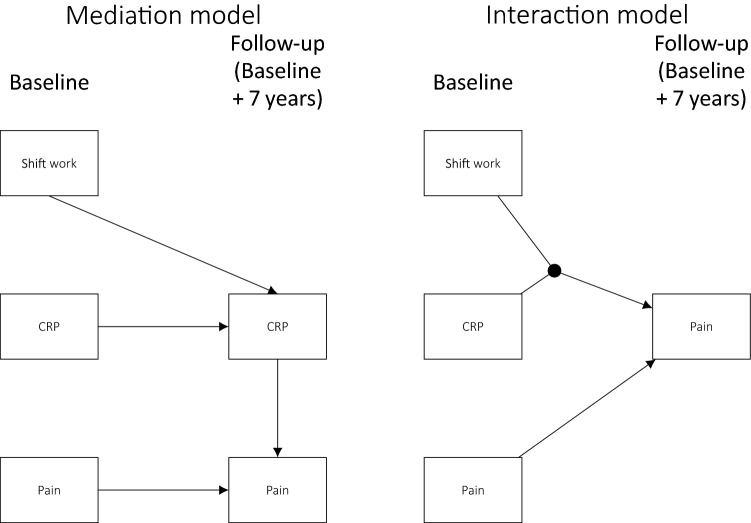
Statistical models: half-longitudinal mediation model and prospective interaction model. Not shown in the figure are the covariates that both CRP and pain at follow-up were regressed on

Analyses were carried out using R version 3.4.4 (R Core Team 2018). An overview of the conceptual models are given in Fig. 1, and an illustration of the statistical models used is given in Fig. 2.

Prior to mediation and interaction analyses, “simple regression analyses” were conducted. That is, prospective regressions were conducted to establish potential relationships between shift work (predictor), CRP (mediator), and pain (outcome), which was analyzed as chronic pain of single pain sites, chronic widespread pain, any chronic pain and as number of chronic pain sites.

For dichotomous outcomes, binary logistic regressions were conducted to estimate relationships of shift work and CRP with “mild” or “severe” chronic pain. The estimates were presented as odds ratios (ORs), comparing the odds of having chronic pain with the odds of not having chronic pain. This was applied to the single pain site variables, “chronic widespread pain”, and “any chronic pain”.

As “number of chronic pain sites” is a count variable, we used Poisson regression for this outcome. Poisson regression estimates were presented as incidence rate ratios (IRRs), comparing the expected count of the dependent variable between levels of the independent variable (e.g., the number of pain sites in shift workers versus non-shift workers).

To estimate the relationship between shift work and CRP ordinal logistic regressions were conducted, as CRP was used as an ordered categorical variable. Estimates were presented as ORs, comparing the odds of higher CRP levels with the odds of lower levels, assuming proportional odds (i.e., that the OR is the same for all cutpoints of the dependent variable). All regressions were adjusted for age, sex, educational level, and prior pain status (i.e., whether or not chronic pain was reported also at baseline), and a *p* value of < 0.05 was considered statistically significant.

### Mediation analyses

In statistical terms, mediation refers to a variable that explains or “carries” the association between two other variables (Lee et al. 2016). Hence, assessing mediation involves an attempt to describe mechanisms and explain why an independent variable (e.g., shift work) predicts a dependent variable (e.g., pain). Hence, in the current case, CRP was considered a potential mediator of the effect of shift work on pain (see Fig. 1).

The statistical test of mediation was conducted with the mediator (CRP) measured at follow‐up, implying that the model estimated the effect of shift work on CRP 7 years later, and furthermore the effect of CRP at follow‐up on pain at follow‐up. Mediation was tested using causal mediation analyses based on counterfactuals (Tingley et al. 2014). This is a flexible approach based on simulations, that allows the use of any type of regression model (e.g., linear, logistic, or Poisson) and allows the accurate decomposition of total effects into indirect and direct effects (Lee et al. 2016; Tingley et al. 2014). Direct effects are effects of the independent variable on the dependent variable that are controlled for the mediator, whereas indirect effects are the effects that go through the mediator (e.g., the effect of shift on CRP and the effect of CRP on pain). Nonparametric bootstrap confidence interval levels were reported, as they reflect a highly reliable way to test the significance of indirect effects by dealing with the non‐normality of the indirect effect (Little 2013). One thousand replications were used for bootstrapping in all analyses.

### Interaction/moderation analyses

Interaction, or moderation, occurs when the level of one variable (a moderator) influences the effect of another variable (independent variable) on a third variable (dependent variable). The independent variable and the moderator are then said to interact in their impact on the dependent variable. This implies a total effect that is larger or smaller than the sum of the effects of the independent variable and the moderator. In the current case, this would imply e.g., that shift work (moderator) potentiated the effect of CRP (independent) on pain (dependent), implying that working shifts with elevated CRP is more likely to induce pain than working regular hours with CRP.

To investigate interaction, the Relative Excess Risk due to Interaction (RERI) (Rothman et al. 2008) was calculated for combinations of working shifts and exhibiting CRP levels at baseline of more than one or 3 mg/L, compared with neither working shifts or exhibiting elevated CRP levels. The RERI can be interpreted as the additional risk that is due to the interaction, expressed as the difference between the effect that would be expected based on summation of separate effects of the risk factors and the observed effect of combined exposure to those risk factors (Jager et al. 2011). A positive RERI (i.e., > 0) indicates positive interaction on an additive scale, implying that the combination of exposures is associated with a stronger effect than the sum of the effects of each separate exposure would imply.

## Results

### Participants

As shown in Table 1, 12.7% of participants had worked shifts at baseline. More than half of participants reported chronic pain in at least one body region (51.9%), while 3.8% reported chronic widespread pain.

Analyses were conducted to determine whether there was selective non‐response after baseline. Since information about subjects that did not participate was unavailable, “non‐response” is in this case defined as a participant not submitting relevant information to the current analyses. As shown in supplementary table S1, age, sex and education were associated with non‐response at baseline. The probability of not responding increased with age (OR: 1.03, 95% CI: 1.02, 1.04) and decreased with higher education, with the lowest likelihood for subjects with a college or university education of more than three years (OR: 0.34, 95% CI: 0.33, 0.56). Males were also less likely not to respond (OR: 0.73, 95% CI: 0.62, 0.87).

Supplementary table S2 shows that age, sex and educational level were also predictive of dropout, i.e. response at baseline but nonresponse at follow‐up (age OR: 1.03, 95% CI: 1.01, 1.05, male OR: 0.77, 95% CI: 0.59, 0.99, college/university > 3 years OR: 0.64, 95% CI: 0.43, 0.98). In addition, chronic neck pain was associated with an increased probability of not responding at follow‐up for baseline participants (OR: 1.46, 95% CI: 1.07, 1.97).

### Simple regression analyses

The simple regression analyses revealed that shift work at baseline was not statistically significantly associated with any of the chronic pain variables 7 years after (Table 2). Moreover, shift workers did not differ from non-shift workers in levels of CRP (supplementary table S3). Cross‐sectional analyses at follow‐up, however, showed that higher CRP levels were associated with higher levels of low back pain (1–2.99 mg/L: OR 1.27, 95% CI 1.02, 1.57, 3–10 mg/L: OR 1.70, 95% CI 1.25, 2.32), hip‐ or leg pain (1–2.99 mg/L: OR 1.30, 95% CI 1.06, 1.59, 3–10 mg/L: OR 1.50, 95% CI 1.11, 2.03), and a higher number of chronic pain sites (3–10 mg/L: IRR 1.17, 95% CI 1.05, 1.30, supplementary table S4).

**Table 2:** Results from separate prospective binary logistic regressions estimating effects of shift work at baseline on reporting mild-to-severe chronic pain of single pain sites at follow‐up 7 years later

Outcome	OR/IRR	95% CI
Neck pain	0.99	(0.75, 1.29)
Arm pain	0.96	(0.71, 1.28)
Upper back pain	0.99	(0.69, 1.41)
Low back pain	1.03	(0.77, 1.37)
Hip or leg pain	0.93	(0.70, 1.23)
Other pain	1.07	(0.63, 1.72)
Any chronic pain	1.41	(0.97, 2.01)
Chronic widespread pain	1.06	(0.59, 1.80)
Number of chronic pain sites	1.16	(0.88, 1.52)

Binary logistic regressions were run for separate pain outcomes, binary logistic regressions were run for ‘any chronic pain’ and ‘chronic widespread pain’, and Poisson regressions were run for ‘number of chronic pain sites’

*OR* odds ratio (for logistic regressions), *IRR* incidence rate ratio (for poisson regressions),* 95% CI* 95% confidence interval

### Mediation

The causal mediation analyses revealed no statistically significant effects (supplementary table S5), indicating no effects of shift work on pain through CRP.

### Interaction analyses

As shown in Table 3, a number of effects were revealed when combining exposure to shift work and elevated CRP, indicating that although shift work did not seem to increase the risk of pain separately, it did add risk when combined with higher CRP. The combination of working shifts and experiencing CRP levels between 1 and 2.99 mg/L was associated with significantly elevated risk of low back pain compared to lower CRP levels combined with no shiftwork (OR: 1.80, 95% CI: 1.14, 2.80). The same comparisons were statistically significant for hip/leg pain (OR: 1.90, 95% CI: 1.22, 2.92), any chronic pain (OR: 1.76, 95% CI: 1.12, 2.85), and number of chronic pain sites (OR: 1.33, 95% CI: 1.14, 1.54). However, for low back pain, hip/leg pain, and number of chronic pain sites a statistically significant effect was observed also for CRP between 1 and 2.99 without shift work (OR: 1.30, 95% CI: 1.04, 1.63, OR: 1.47, 95% CI: 1.18, 1.83, and OR: 1.13, 95% CI: 1.22, respectively). Moreover, CRP > 3 mg/L combined with not working shifts was associated with increased risk of arm pain (OR: 1.42, 95% CI: 1.03, 1.96) and low back pain (OR: 1.46, 95% CI: 1.06, 2.00).

**Table 3:** odds ratios comparing the likelihood of chronic pain at follow-up for different combinations of shiftwork and level of CRP at baseline 7 years prior

	CRP < 1	CRP 1‐2.99	CRP > 2.99	RERI
	OR/IRR^a^ (95% CI)	OR/IRR^a^ (95% CI)	OR/IRR^a^ (95% CI)	Est (95% CI)
Neck pain				
No shiftwork	Ref ‐	1.04 (0.84,1.28)	1.01 (0.74, 1.37)	0.56 (− 0.12, 1.24)
Shiftwork	0.84 (0.57, 1.22)	1.43 (0.93, 2.22)	0.69 (0.31, 1.47)	–
Arm pain				
No shiftwork	Ref ‐	1.24 (0.98, 1.56)	1.42 (1.03, 1.96)^c^	0.25 (− 0.49, 0.99)
Shiftwork	0.91 (0.59, 1.37)	1.40 (0.87, 2.21)	1.05 (0.45, 2.28)	–
Upper back pain				
No shiftwork	Ref ‐	1.20 (0.91, 1.58)	1.11 (0.74, 1.63)	0.14 (− 0.76, 1.04)
Shiftwork	1.01 (0.60, 1.64)	1.35 (0.75, 2.33)	0.69 (0.19, 1.90)	–
Low back pain No shiftwork	Ref ‐	1.30 (1.04, 1.63)^c^	1.46 (1.06, 2.00)^c^	0.57 (− 0.29, 1.43)
Shiftwork	0.93 (0.61, 1.39)	1.80 (1.14, 2.80)^c^	0.99 (0.42, 2.16)	–
Hip/leg pain No shiftwork	Ref ‐	1.47 (1.18, 1.83)^c^	1.36 (0.99, 1.85)	0.63 (− 0.23, 1.49)
Shiftwork	0.80 (0.52, 1.20)	1.90 (1.22, 2.92)^c^	0.81 (0.34, 1.77)	–
Other pain				
No shiftwork	Ref ‐	1.46 (0.97, 2.21)	1.31 (0.71, 2.29)	− 0.08 (− 1.52, 1.35)
Shiftwork	1.17 (0.54, 2.31)	1.55 (0.68, 3.20)	1.05 (0.16, 3.71)	–
Chronic				
Widespread pain				
No shiftwork	Ref ‐	1.44 (0.92, 2.26)	0.89 (0.42, 1.74)	− 0.64 (− 2.32, 1.04)
Shiftwork	1.57 (0.72, 3.15)	1.37 (0.52, 3.15)	_‐_b_‐_b	–
Any chronic pain No shiftwork	Ref ‐	1.20 (0.97, 1.49)	1.19 (0.88, 1.61)	0.87 (0.02, 1.71)^c^
Shiftwork	0.69 (0.48, 1.00)	1.76 (1.12, 2.85)^c^	1.14 (0.54, 2.50)	–
Number of chronic pain sites				
No shiftwork	Ref ‐	1.13 (1.05, 1.22)^c^	1.12 (1.00, 1.24)	0.26 (0.02, 0.49)^c^
Shiftwork	0.94 (0.81, 1.08)	1.33 (1.14, 1.54)^c^	0.91 (0.67, 1.20)	–

Binary logistic regressions were run single pain sites, chronic widespread pain, and any chronic pain as outcome, and poisson regression for number of chronic pain sites as outcome

All regressions were adjusted For sex, age, educational level, and the respective outcome at baseline

^a^For analyses with number of chronic pain sites as outcome, IRRs were computed

*95% CI* 95% confidence interval, *OR* odds ratio, *IRR* incidence rate ratio

^b^The number of subjects in this category was too small to allow computation of effects

^c^*p* < 0.05

The calculation of the RERI revealed that for any chronic pain and number of chronic pain sites the effects of combined exposure appeared to be synergistic (estimates 0.87, 95% CI 0.02, 1.71 and 0.26, 95% CI 0.02, 0.49, respectively), suggesting that the risks of any chronic pain or a higher number of chronic pain sites after shift work were exacerbated for subjects with higher CRP levels.

## Discussion

The aim of the present study was to elucidate the prospective, long term relationship of shift work with chronic pain by investigating the role of CRP in determining this relationship. However, our current analyses provided little evidence of shift work predicting chronic pain complaints or CRP levels seven years subsequently. Not surprisingly, then, no evidence was observed of CRP mediating effects of shift work on chronic pain (see table S5). However, associations between CRP and chronic pain complaints were observed for low back pain, hip/leg pain, and number of chronic pain sites (Table S2). In conclusion, while CRP was, to some extent, linked to pain, shift work did not appear to affect CRP or pain. Hence, the notion that shift work promotes low‐grade systemic inflammation, which in turn promotes chronic pain, received no support from the current analyses.

Interestingly, however, for some outcome measures, the combined exposure to shift work and higher levels of CRP was associated with elevated risk of chronic pain (see Table 3). Specifically, for “any chronic pain”, the combination of shift work and higher CRP levels was associated with elevated risk. These two exposures were not statistically significantly associated with “any chronic pain” separately. Hence, the results suggest that the exposure load of either shift work or higher levels of CRP alone was not sufficient to produce or maintain chronic pain. Additionally, for “any chronic pain” and “number of chronic pain sites”, a departure from additivity was also apparent. This suggests that not only is the combination of exposures sometimes required to produce an effect on pain, but the aversive impact of shift work may also exacerbate the pain‐promoting effects of inflammation.

Seemingly in disagreement with the current results, a previous study by Puttonen and coworkers (Puttonen et al 2011) reported associations of shift work with CRP. However, those associations were evident only for shift work that included night work, and predominantly for males. An association has also between suggested between number of years in shiftwork, a variable not available in the present data, and CRP (Skogstad et al 2019).

With regards to the association between shift work and musculoskeletal pain, the present results are in line with a recent cross‐sectional study, which found that pain complaints did not differ between shift workers and day only workers (Matre et al. 2020). However, at least two longitudinal studies support the hypothesis that shift workers are at higher risk for developing low back pain (Zhao et al. 2012), or for developing pain in the neck, shoulder or back (Trinkoff et al. 2006). Differences in assessment of both exposure and outcome may have contributed to the divergent results. Also, an experimental study of nurses demonstrated increased pain sensitivity after consecutive night shifts (Matre et al. 2017). This points to a limitation of the current exposure measure, as it was quite general and did not allow distinctions between types of shifts worked or characteristics of the work being carried out. This non‐specificity may have resulted in a failure to detect true associations. Hence, future studies should aim to include more detailed measures of exposure in order to investigate effects of different types of shift as well as number of consecutive shifts, quick returns, and other aspects of shift work that may affect the extent to which it influences health.

The current analyses showed that CRP was only associated with some types of pain, i.e., low back, hip/leg, and number of chronic pain sites. Hence, it remains uncertain to what extent the measurement of non‐clinical levels of circulating CRP represents a valid model for inflammatory pain. Recent systematic reviews have only partly supported the notion of CRP being a marker of specific pain conditions, for instance low back pain (e.g., see Berg et al. 2018; Lim et al. 2020).

The current study investigated chronic pain. This should be kept firmly in mind, as the possibility remains that transitory pain is affected by shift work (Matre et al. 2020). Episodic and recurring pain may represent considerable health problems for the individual and high costs for society even when criteria for chronic pain are not satisfied.

### Methodological considerations

Although a number of strengths of the current study can be noted, such as the large population sample and the long follow‐up period, some considerations should be kept in mind when interpreting the results. The most notable limitation was the use of a single item self‐reported measure of shift work, as discussed above. For instance, in a review of health consequences of shift work, Kecklund and Axelsson (2016) pointed out that adverse health effects seemed to be primarily associated with night work, as opposed to shifts limited to day or evening work. Hence, the lack of information about shift type may have obscured true associations. Moreover, as is often the case in prospective cohort studies, shift work was assessed only at baseline, hence the course of exposure during the follow-up period remains unknown. The accumulated number of years working shifts may influence CRP levels (Skogstad et al. 2019). If many of the shift workers of the current sample stopped working shifts during the follow-up period, effects may have been diluted, and may perhaps be more representative of long-term, lagged effects of shift work rather than effects of long-term exposure to shift work.

Measuring both shift work and pain by subjective self‐report may increase the likelihood of self‐report bias influencing both exposure and outcome measures, which could induce common method bias (Podsakoff et al. 2003). However, these measures were recorded 7 years apart, hence the probability of situationally specific factors (“occasion factors”), such current mood and life circumstances influencing the self-report at both time points is not as high as when measures are recorded temporally close (Podsakoff et al. 2003). Furthermore, while the influence of mood and negative affect on pain reporting is plausible, it seems less likely that emotional states would cause subjects to misjudge whether or not they have worked shifts the previous three months. Finally, if negative emotional states were induced or exacerbated by shift work and circadian stress, and subsequently lead to pain, this would represent a substantive mechanism rather than a bias.

Healthy worker effects seem particularly plausible in the current study design—after 7 years—workers with chronic pain may tend to exit the workforce, change to jobs that are less aversive to their conditions, or reduce the number of hours at work (Breivik et al. 2006). This implies that it is difficult to detect true associations over such a long time period. Employees that participated were in full time employment at both measurement points, but it remains unknown whether they changed jobs during the follow‐up period, which may imply alleviation of exposure and perhaps substantially diluted effect estimates. Moreover, although analyses were adjusted for pain complaints at baseline, participants’ complete medical history was not available. Unknown medical conditions, such as e.g. cardiovascular disease, may have caused some participants to avoid shift work, while exhibiting elevated CRP and pain symptoms. If so, associations of shift with CRP and pain may have been under-estimated.

The long follow‐up period should be a strength as it allows the detection of long-term health problems. However, as no clear assumption can be made about a pathogenic mechanism, it remains unknown whether it is in fact appropriate. Seven years may be overly conservative, especially if the abovementioned healthy worker selection mechanisms are at play. Moreover, for workers with chronic pain, varying degrees of sick leave and treatment could have occurred and resulted in improved health before the end of the 7 years period.

A relatively high number of tests were conducted in the current research. Hence, the possibility cannot be excluded that some results appeared due to chance. Considering the relatively limited findings of effects of shiftwork in the current study, replication should be a priority for future studies.

## Concluding remarks

The current results suggested limited long‐term effects of shift work on chronic pain complaints, and no evidence was found of CRP mediating such effects. Chronic pain is multifactorial and the pain experienced by shift workers in the current healthy sample was not predominantly due to processes associated with levels of CRP. However, the analyses suggested that, at least for some types of chronic pain, shift work may exacerbate (i.e., interact with) the effects of low‐grade inflammation. Hence, working shifts may promote the experience of pain for those already exhibiting higher levels of CRP. This should be equally relevant to researchers, practitioners, and policy makers, as it suggests workers already exhibiting high levels of CRP should avoid shift work, even if not experiencing pain symptoms. Future research should take into account more detailed aspects of shift work with varying time lags in order to further elucidate the effects that the current study provided preliminary evidence of.

## Supplementary Information

Below is the link to the electronic supplementary material.

Supplementary file1 (DOCX 34 KB)

## Data Availability

The permission to analyze data was given by the Arctic University of Norway in Tromsø. Data cannot be made publicly available, but may be available upon request.
