# Supplementary material for: Shift work, low-grade inflammation, and chronic pain: a 7-year prospective study
Source: Int Arch Occup Environ Health. 2021 Feb 7;94(5):1013–22. doi: 10.1007/s00420-020-01626-2 (PMC8238752; doi:10.1007/s00420-020-01626-2)
Supplement: Supplementary file 1 — Supplementary file1 (DOCX 34 KB) [file 420_2020_1626_MOESM1_ESM.docx]

| Table S1. *Non‐response analyses: Binary logistic regression with non‐response (i.e. incomplete data) at baseline regressed on education, sex, and age (N = 3318)* | | |
| --- | --- | --- |
| **Predictor** | **OR** | **95% CI** |
| Age | 1.03 | [1.02,1.04]^*^ |
| Sex |  |  |
| Female | Ref |  |
| Male | 0.73 | [0.62,0.87]^*^ |
| Education |  |  |
| Primary/secondary school | Ref |  |
| Technical/vocational school,  1‐2 years senior high school | 0.75 | [0.57,0.98]^*^ |
| High school diploma | 0.62 | [0.44,0.87]^*^ |
| College/university <4 years | 0.46 | [0.35,0.62]^*^ |
| College/university >3 years | 0.34 | [0.33,0.56]^*^ |

* p<0.05, OR: Odds ratio, 95% CI: 95% confidence interval

| Table S2. *Attrition analyses: Binary logistic regression with non‐response (i.e. incomplete data) at follow‐up regressed on baseline study variables for subjects with complete data at baseline (N = 2613)* | | |
| --- | --- | --- |
|  | OR | 95% CI |
| HS‐CRP | 1.00 | [0.94,1.04] |
| Shift work during the previous 3 months | 1.29 | [0.83,1.70] |
| Age | 1.03 | [1.01,1.05]^*^ |
| Sex |  |  |
| Female | Ref | ‐ |
| Male | 0.77 | [0.59,0.99]^*^ |
| Education |  |  |
| Primary/secondary school | Ref | ‐ |
| Technical/vocational school, |  |  |
| 1‐2 years senior high school | 0.80 | [0.52,1.22] |
| High school diploma | 0.55 | [0.31,0.95]^*^ |
| College/university <4 years | 0.72 | [0.47,1.11] |
| College/university >3 years | 0.64 | [0.43,0.98]^*^ |
| Chronic pain complaints |  |  |
| Neck pain | 1.46 | [1.07,1.97]^*^ |
| Arm pain | 0.88 | [0.63,1.23] |
| Upper back pain | 1.11 | [0.77,1.59] |
| Low back pain | 1.29 | [0.96,1.74] |
| Hip and leg pain | 1.14 | [0.84,1.55] |
| Other pain | 0.68 | [0.33,1.27] |

*p<0.05, OR: Odds ratio, 95% CI: 95% confidence interval

| Table S3. *Prospective ordered logistic regression estimating the effect of shift work on CRP seven years later* | |
| --- | --- |
|  | OR (95% CI) |
| Shift work baseline *→* CRP follow‐up | 1.24 (0.94,1.62) |
| *p<0.05, OR: Odds ratio, 95% CI: 95% confidence interval  The regression was adjusted for sex, age, educational level, and CRP at baseline | |

| Table S4. *Results from separate cross‐sectional regressions estimating effects of CRP on chronic pain at follow‐up* | | | |
| --- | --- | --- | --- |
| Outcome | CRP | OR/IRR^a^ | 95% CI |
| Neck pain | <1 | Ref | ‐ |
|  | 1 ‐ 2.99 | 1.07 | [0.87,1.31] |
|  | 3 ‐ 10 | 1.03 | [0.76,1.40] |
| Arm pain | <1 | Ref | ‐ |
|  | 1 ‐ 2.99 | 1.08 | [0.87,1.35] |
|  | 3 ‐ 10 | 1.26 | [0.91,1.73] |
| Upper back pain | <1 | Ref | ‐ |
|  | 1 ‐ 2.99 | 1.02 | [0.78,1.33] |
|  | 3 ‐ 10 | 1.44 | [0.99,2.07] |
| Low back pain | <1 | Ref | ‐ |
|  | 1 ‐ 2.99 | 1.27 | [1.02,1.57]^*^ |
|  | 3 ‐ 10 | 1.70 | [1.25,2.32]^*^ |
| Hip or leg pain | <1 | Ref | ‐ |
|  | 1 ‐ 2.99 | 1.30 | [1.06,1.59]^*^ |
|  | 3 ‐ 10 | 1.50 | [1.11,2.03]^*^ |
| Other pain | <1 | Ref | ‐ |
|  | 1 ‐ 2.99 | 1.02 | [0.68,1.52] |
|  | 3 ‐ 10 | 1.59 | [0.94,2.61] |
| Any | <1 | Ref | ‐ |
| chronic pain | 1 ‐ 2.99 | 1.21 | [0.99,1.48] |
|  | 3 ‐ 10 | 1.30 | [0.96,1.78] |
| Chronic | <1 | Ref | ‐ |
| widespread pain | 1 ‐ 2.99 | 1.20 | [0.79,1.81] |
|  | 3 ‐ 10 | 0.61 | [0.27,1.24] |
| Number of chronic pain sites | <1 | Ref | ‐ |
|  | 1 ‐ 2.99 | 1.07 | [1.00,1.15] |
|  | 3 ‐ 10 | 1.17 | [1.05,1.30]^*^ |
| *p<0.05, 95% CI: 95% confidence interval, OR: Odds ratio (for logistic regressions), IRR: Incidence rate ratio (for poisson regressions)  Binary logistic regressions were run for separate pain outcomes, ’any chronic pain’, and ’chronic widespread pain’, and poisson regressions were run for ’number of chronic pain sites’ All regressions were adjusted for sex, age, and educational level. | | | |

| Table S5. Results from mediation analyses of halflongitudinal mediation models with chronic pain in single pain sites, in any pain site, and widespread chronic pain as outcomes and shiftwork as exposure | | | | | | |
| --- | --- | --- | --- | --- | --- | --- |
|  | Direct effect | | Indirect effect | | Total effect | |
|  | Estimate | 95% CI | Estimate | 95% CI | Estimate | 95% CI |
| Neck pain | 0.008 | [-0.049,0.063] | 0.000 | [-0.002,0.002] | 0.008 | [-0.049,0.063] |
| Arm pain | -0.008 | [-0.059,0.046] | 0.001 | [-0.001,0.004] | -0.007 | [-0.059,0.046] |
| Upper back pain | 0.003 | [-0.037,0.047] | 0.001 | [-0.001,0.003] | 0.004 | [-0.037,0.047] |
| Low back pain | 0.002 | [-0.049,0.055] | 0.003 | [-0.001,0.007] | 0.005 | [-0.047,0.059] |
| Hip or leg pain | -0.012 | [-0.062,0.041] | 0.002 | [-0.001,0.007] | -0.010 | [-0.060,0.044] |
| Other pain | 0.004 | [-0.025,0.032] | 0.000 | [-0.001,0.002] | 0.004 | [-0.025,0.032] |
| Chronic widespread pain | 0.008 | [-0.017,0.032] | 0.000 | [-0.002,0.001] | 0.008 | [-0.017,0.033] |
| Any chronic pain | -0.013 | [-0.069,0.043] | 0.002 | [-0.001,0.006] | -0.011 | [-0.067,0.045] |
| Number of chronic pain sites | 0.025 | [-0.132,0.189] | 0.007 | [-0.003,0.020] | 0.031 | [-0.125,0.197] |

*p<0.05, 95% CI: 95% confidence interval

Confidence intervals were bootstrapped with 1000 draws for each analysis

All analyses were adjusted for sex, age, educational level, and the respective outcome at baseline.

| Table S1. *Non‐response analyses: Binary logistic regression with non‐response (i.e. incomplete data) at baseline regressed on education, sex, and age (N = 3318)* | | |
| --- | --- | --- |
| **Predictor** | **OR** | **95% CI** |
| Age | 1.03 | [1.02,1.04]^*^ |
| Sex |  |  |
| Female | Ref |  |
| Male | 0.73 | [0.62,0.87]^*^ |
| Education |  |  |
| Primary/secondary school | Ref |  |
| Technical/vocational school,  1‐2 years senior high school | 0.75 | [0.57,0.98]^*^ |
| High school diploma | 0.62 | [0.44,0.87]^*^ |
| College/university <4 years | 0.46 | [0.35,0.62]^*^ |
| College/university >3 years | 0.34 | [0.33,0.56]^*^ |

* p<0.05, OR: Odds ratio, 95% CI: 95% confidence interval

| Table S2. *Attrition analyses: Binary logistic regression with non‐response (i.e. incomplete data) at follow‐up regressed on baseline study variables for subjects with complete data at baseline (N = 2613)* | | |
| --- | --- | --- |
|  | OR | 95% CI |
| HS‐CRP | 1.00 | [0.94,1.04] |
| Shift work during the previous 3 months | 1.29 | [0.83,1.70] |
| Age | 1.03 | [1.01,1.05]^*^ |
| Sex |  |  |
| Female | Ref | ‐ |
| Male | 0.77 | [0.59,0.99]^*^ |
| Education |  |  |
| Primary/secondary school | Ref | ‐ |
| Technical/vocational school, |  |  |
| 1‐2 years senior high school | 0.80 | [0.52,1.22] |
| High school diploma | 0.55 | [0.31,0.95]^*^ |
| College/university <4 years | 0.72 | [0.47,1.11] |
| College/university >3 years | 0.64 | [0.43,0.98]^*^ |
| Chronic pain complaints |  |  |
| Neck pain | 1.46 | [1.07,1.97]^*^ |
| Arm pain | 0.88 | [0.63,1.23] |
| Upper back pain | 1.11 | [0.77,1.59] |
| Low back pain | 1.29 | [0.96,1.74] |
| Hip and leg pain | 1.14 | [0.84,1.55] |
| Other pain | 0.68 | [0.33,1.27] |

*p<0.05, OR: Odds ratio, 95% CI: 95% confidence interval

| Table S3. *Prospective ordered logistic regression estimating the effect of shift work on CRP seven years later* | |
| --- | --- |
|  | OR (95% CI) |
| Shift work baseline *→* CRP follow‐up | 1.24 (0.94,1.62) |
| *p<0.05, OR: Odds ratio, 95% CI: 95% confidence interval  The regression was adjusted for sex, age, educational level, and CRP at baseline | |

| Table S4. *Results from separate cross‐sectional regressions estimating effects of CRP on chronic pain at follow‐up* | | | |
| --- | --- | --- | --- |
| Outcome | CRP | OR/IRR^a^ | 95% CI |
| Neck pain | <1 | Ref | ‐ |
|  | 1 ‐ 2.99 | 1.07 | [0.87,1.31] |
|  | 3 ‐ 10 | 1.03 | [0.76,1.40] |
| Arm pain | <1 | Ref | ‐ |
|  | 1 ‐ 2.99 | 1.08 | [0.87,1.35] |
|  | 3 ‐ 10 | 1.26 | [0.91,1.73] |
| Upper back pain | <1 | Ref | ‐ |
|  | 1 ‐ 2.99 | 1.02 | [0.78,1.33] |
|  | 3 ‐ 10 | 1.44 | [0.99,2.07] |
| Low back pain | <1 | Ref | ‐ |
|  | 1 ‐ 2.99 | 1.27 | [1.02,1.57]^*^ |
|  | 3 ‐ 10 | 1.70 | [1.25,2.32]^*^ |
| Hip or leg pain | <1 | Ref | ‐ |
|  | 1 ‐ 2.99 | 1.30 | [1.06,1.59]^*^ |
|  | 3 ‐ 10 | 1.50 | [1.11,2.03]^*^ |
| Other pain | <1 | Ref | ‐ |
|  | 1 ‐ 2.99 | 1.02 | [0.68,1.52] |
|  | 3 ‐ 10 | 1.59 | [0.94,2.61] |
| Any | <1 | Ref | ‐ |
| chronic pain | 1 ‐ 2.99 | 1.21 | [0.99,1.48] |
|  | 3 ‐ 10 | 1.30 | [0.96,1.78] |
| Chronic | <1 | Ref | ‐ |
| widespread pain | 1 ‐ 2.99 | 1.20 | [0.79,1.81] |
|  | 3 ‐ 10 | 0.61 | [0.27,1.24] |
| Number of chronic pain sites | <1 | Ref | ‐ |
|  | 1 ‐ 2.99 | 1.07 | [1.00,1.15] |
|  | 3 ‐ 10 | 1.17 | [1.05,1.30]^*^ |
| *p<0.05, 95% CI: 95% confidence interval, OR: Odds ratio (for logistic regressions), IRR: Incidence rate ratio (for poisson regressions)  Binary logistic regressions were run for separate pain outcomes, ’any chronic pain’, and ’chronic widespread pain’, and poisson regressions were run for ’number of chronic pain sites’ All regressions were adjusted for sex, age, and educational level. | | | |

| Table S5. Results from mediation analyses of halflongitudinal mediation models with chronic pain in single pain sites, in any pain site, and widespread chronic pain as outcomes and shiftwork as exposure | | | | | | |
| --- | --- | --- | --- | --- | --- | --- |
|  | Direct effect | | Indirect effect | | Total effect | |
|  | Estimate | 95% CI | Estimate | 95% CI | Estimate | 95% CI |
| Neck pain | 0.008 | [-0.049,0.063] | 0.000 | [-0.002,0.002] | 0.008 | [-0.049,0.063] |
| Arm pain | -0.008 | [-0.059,0.046] | 0.001 | [-0.001,0.004] | -0.007 | [-0.059,0.046] |
| Upper back pain | 0.003 | [-0.037,0.047] | 0.001 | [-0.001,0.003] | 0.004 | [-0.037,0.047] |
| Low back pain | 0.002 | [-0.049,0.055] | 0.003 | [-0.001,0.007] | 0.005 | [-0.047,0.059] |
| Hip or leg pain | -0.012 | [-0.062,0.041] | 0.002 | [-0.001,0.007] | -0.010 | [-0.060,0.044] |
| Other pain | 0.004 | [-0.025,0.032] | 0.000 | [-0.001,0.002] | 0.004 | [-0.025,0.032] |
| Chronic widespread pain | 0.008 | [-0.017,0.032] | 0.000 | [-0.002,0.001] | 0.008 | [-0.017,0.033] |
| Any chronic pain | -0.013 | [-0.069,0.043] | 0.002 | [-0.001,0.006] | -0.011 | [-0.067,0.045] |
| Number of chronic pain sites | 0.025 | [-0.132,0.189] | 0.007 | [-0.003,0.020] | 0.031 | [-0.125,0.197] |

*p<0.05, 95% CI: 95% confidence interval

Confidence intervals were bootstrapped with 1000 draws for each analysis

All analyses were adjusted for sex, age, educational level, and the respective outcome at baseline.
